# Relationship between individual chamber and whole shell Mg/Ca ratios in *Trilobatus sacculifer* and implications for individual foraminifera palaeoceanographic reconstructions

**DOI:** 10.1038/s41598-020-80673-8

**Published:** 2021-01-11

**Authors:** Gerald T. Rustic, Pratigya J. Polissar, Ana Christina Ravelo, Peter DeMenocal

**Affiliations:** 1 Department of Geology, School of Earth and Environment, Rowan University, Glassboro, NJ 08028 USA; 2 Lamont-Doherty Earth Observatory, Columbia University, Palisades, NY 19604 USA; 3 Ocean Sciences Department, University of California at Santa Cruz, Santa Cruz, CA 95064 USA; 4 Woods Hole Oceanographic Institution, Woods Hole, MA 02543 USA

**Keywords:** Palaeoceanography, Palaeoclimate

## Abstract

Precisely targeted measurements of trace elements using laser ablation inductively coupled plasma mass spectrometry (LA-ICPMS) reveal inter-chamber heterogeneities in specimens of the planktic foraminifer *Trilobatus (Globigerinoides) sacculifer*. We find that Mg/Ca ratios in the final growth chamber are generally lower compared to previous growth chambers, but final chamber Mg/Ca is elevated in one of thirteen sample intervals. Differences in distributions of Mg/Ca values from separate growth chambers are observed, occurring most often at lower Mg/Ca values, suggesting that single-chamber measurements may not be reflective of the specimen’s integrated Mg/Ca. We compared LA-ICPMS Mg/Ca values to paired, same-individual Mg/Ca measured via inductively coupled plasma optical emission spectrometry (ICP-OES) to assess their correspondence. Paired LA-ICPMS and ICP-OES Mg/Ca show a maximum correlation coefficient of R = 0.92 (p < 0.05) achieved by applying a weighted average of the last and penultimate growth chambers. Population distributions of paired Mg/Ca values are identical under this weighting. These findings demonstrate that multi-chamber LA-ICPMS measurements can approximate entire specimen Mg/Ca, and is thus representative of the integrated conditions experienced during the specimen’s lifespan. This correspondence between LA-ICPMS and ICP-OES data links these methods and demonstrates that both generate Mg/Ca values suitable for individual foraminifera palaeoceanographic reconstructions.

## Introduction

The relationship between calcification temperature and Mg/Ca ratios in the shells (or tests) of foraminifera has long been used in paleoclimate reconstructions^1–3^. This relationship has been quantified from core-top, sediment trap, and culture studies, resulting in various single-species and multi-species paleotemperature calibration equations relating temperature to shell Mg/Ca^4–6^. Typically, sea surface temperature (SST) reconstructions generated from Mg/Ca ratios are obtained from monospecific aggregates of 5–50 shells. More recently, the analysis of Mg/Ca ratios from individual foraminifera has opened new dimensions in paleoclimate reconstructions, allowing for palaeoceanographic reconstruction of ocean variability^7–9^. Highly detailed analysis of Mg/Ca ratios within single chambers of individual foraminifera specimens and through the shell layers is possible using laser ablation inductively coupled plasma mass spectrometry (LA-ICPMS)^9–13^. Advances in analytical techniques now also allow measurement of Mg/Ca ratios from individual foraminifera using ICP-MS and ICP-optical emission spectrometry (ICP-OES) through modification of solution chemistry techniques for analyzing larger 5–50 shell samples^14–16^. Inter-method comparisons studies comparing average Mg/Ca obtained from LA-ICPMS on discrete chambers and ICP-MS or ICP-OES analysis of complete shells have found that population mean values are consistent^11,17,18^. However, the correspondence of Mg/Ca values between these methods at the individual level is poorly quantified, and the degree to which variability within foraminifera shells may influence this correspondence is unclear.

In this study we examine Mg/Ca variability within shells of the planktic foraminifer *Trilobatus (Globigerinoides) sacculifer* (^19^, Brady 1877) using LA-ICPMS, and then quantify the relationship between Mg/Ca values obtained from LA-ICPMS and from ICP-OES on the same foraminifera specimens. This approach allows direct comparison of foraminifera Mg/Ca values from these methods at the individual level, and determination of how intrashell Mg/Ca patterns shape the whole shell Mg/Ca value.

## Background

Mg/Ca ratios from individual foraminifera have been used to asses oceanic conditions and variability, including assessment of changes in interannual variability associated with the El Niño Southern Oscillation (ENSO)^7–10,16,20,21^. The shell chemistry of each foraminifer reflects the ocean conditions it experienced during its 2–4 week life span^22^. These ~ monthly ‘snapshots’ generate a distribution of conditions that occurred during the duration of an accumulating sediment interval. Paleoclimatic interpretation of individual foraminifera Mg/Ca data involves analysis of dispersion statistics (e.g., variance, standard deviation, kernel density functions or median absolute deviation) and differences in population distributions^7–9,16,23^ in order to infer the changes in past conditions. Studies using Mg/Ca from individual foraminifera analysis have used both ICP-MS^23^, ICP-OES^16^, and LA-ICPMS^7–9,11^ analytical techniques. While differing analytical methods may provide similar population mean Mg/Ca results, this is no guarantee that the population distributions are similar. For example, the small shell volume sampled by LA-ICPMS could systematically differ from the whole shell, while solution cleaning methods for ICP-MS could remove shell material that is included in LA-ICPMS analysis. At present, the degree to which analytical technique may influence the Mg/Ca values, and thus existing Mg/Ca temperature calibrations, has not been evaluated on individual foraminifera.

Solution-chemistry techniques used for the analysis of Mg/Ca from multiple aggregated specimens involve crushing, washing, and chemical cleaning to remove trace metal contaminants, crusts, and clay minerals^14,24–26^. Modified cleaning procedures that preserve foraminifera calcite for analysis have been developed for use on individual foraminifera^14,15^. Prior to analysis via either ICP-MS or ICP-OES, the cleaned foraminifer is dissolved, and thus the resulting Mg/Ca ratio is an integrated signal from the entire shell, or portions of the shell that remain after chemical cleaning, which can reduce shell mass by up to 73% in specimens of *T. sacculifer*^27^.

Individual foraminifera LA-ICPMS analysis differs from “whole specimen” analysis in that it measures elemental abundances of Mg, Ca, and common contaminant species (Mn, Al) in a precisely targeted region of the foraminifera test, leaving much of the remaining shell intact. This highly-targeted analysis can identify regions of varying composition, including surface contaminant crusts^10,17,28^, outer gametogenic crusts and inner ontogenetic crusts and growth layers^10,29,30^, and depth profiles that suggest diurnal changes in photosynthetic symbiont activity^12,13,31^. Inter-chamber Mg/Ca differences on the same individual are also observed in species commonly used for paleoclimate reconstructions^10,11,29,32,33^. The details of LA-ICPMS analysis can also vary, with some LA-ICPMS studies targeting the final, excised chamber of the mixed-layer foraminifer *T. sacculifer*^7,8,11,34^, and others analyzing multiple chambers of planktic^21,35^ or benthic species^36^. The LA-ICPMS Mg/Ca value for an individual is thus dependent on the specific chamber or chambers and ablation depth, in contrast to the integrated “whole specimen” Mg/Ca obtained via solution chemistry methods and ICP-MS/ICP-OES.

The extent to which inter- and intra-chamber variability observed in individual foraminifera affects palaeoceanographic interpretations of individual foraminifera data is not well constrained. Here we report on intra-shell variability of the mixed-layer dwelling foraminifera *T. sacculifer* from a study site in the central tropical Pacific Ocean. We assess the inter-chamber variability of the final three pre-gametogenic growth chambers of *T. sacculifer* using LA-ICPMS, and use paired, same-shell solution-based chemistry and ICP-OES to assess the relationship between Mg/Ca values from each chamber to that obtained from analysis of the entire foraminifer via ICP-OES.

## Results

### Analysis of LA-ICPMS within-sample chamber populations

**Figure 1:**
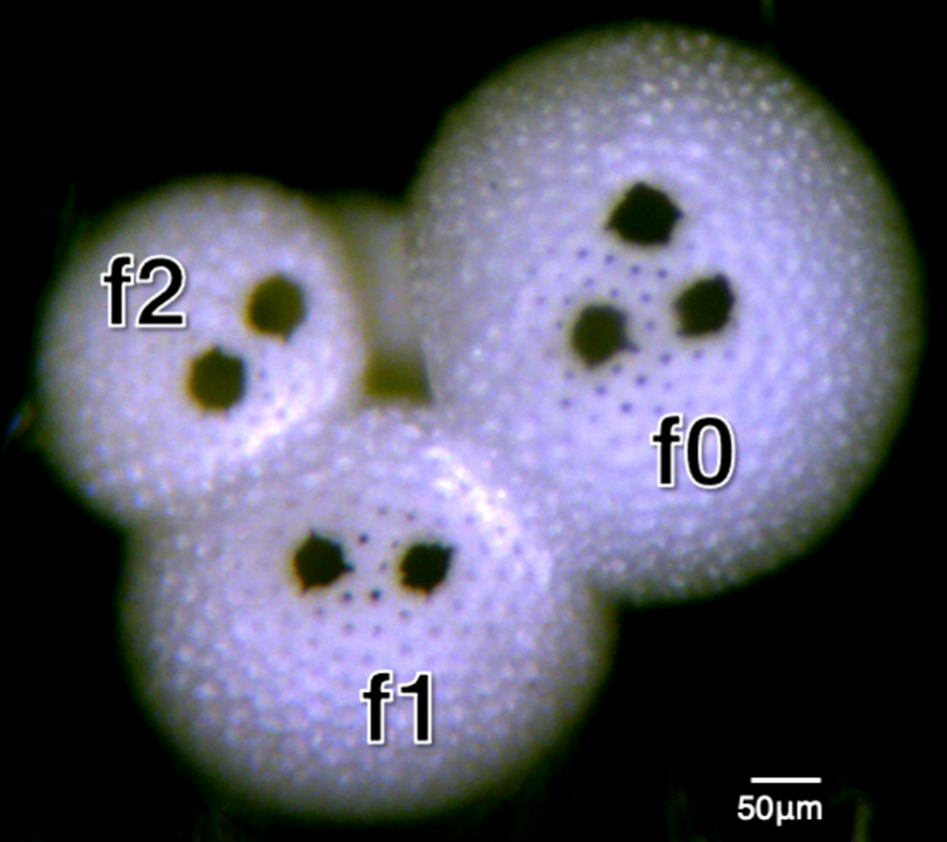
Specimen of *T. sacculifer* after LA-ICPMS showing growth chambers and laser ablation sites. The f0 chamber is the final growth chamber prior to formation of the final sac, f1 is the penultimate chamber, and f2 precedes it. Ablation targets are 50 μm.

**Figure 2:**
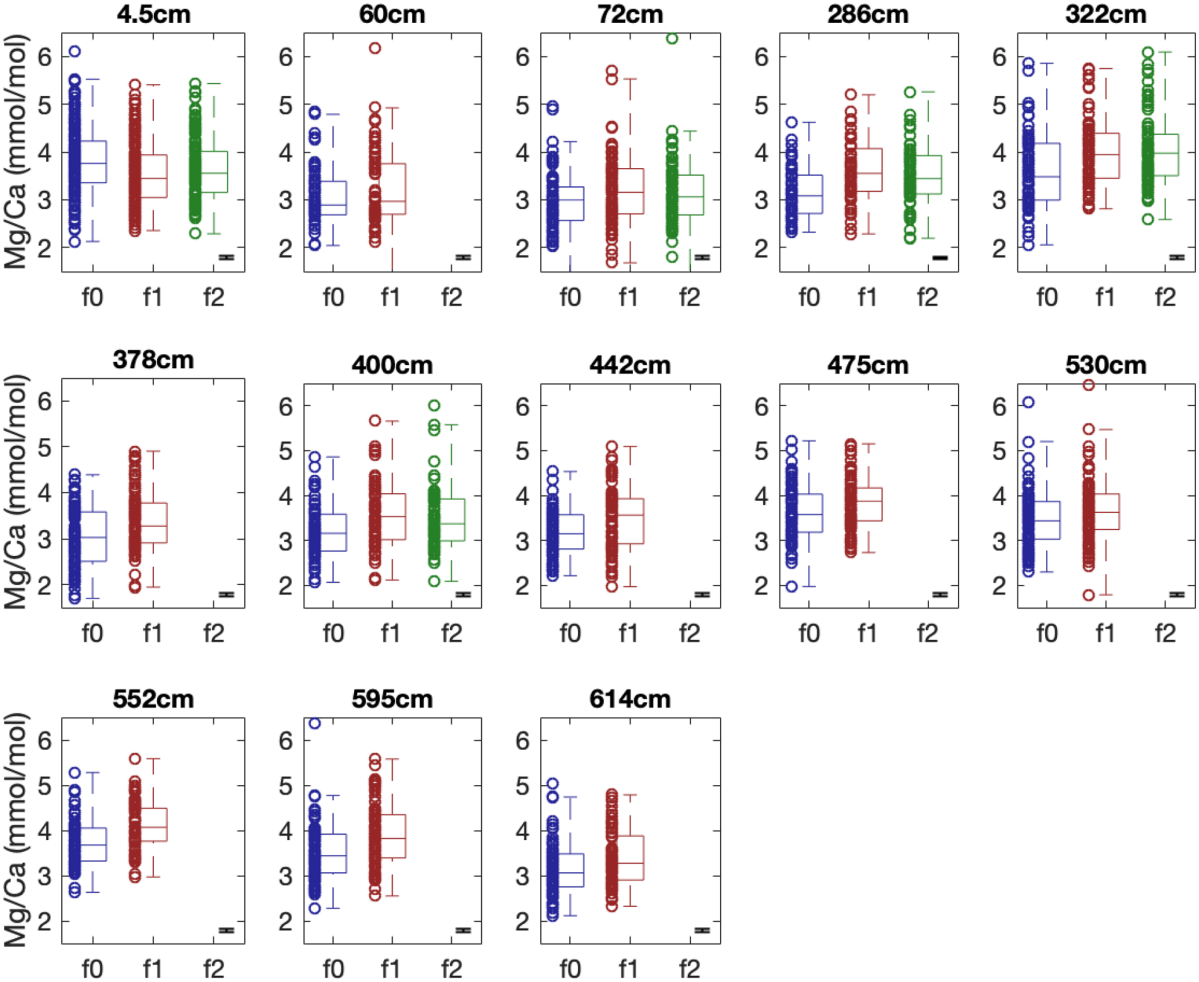
Single specimen Mg/Ca values for each chamber from various sample depths. Each circle is the mean of all laser-ablation ICP-MS (LA-ICPMS) Mg/Ca measurements on one chamber from a single specimen. The error bar at the right of each plot shows the mean analytical uncertainty (the standard error of the mean of individual laser ablation measurements on a given chamber) from that interval. Boxes show the interquartile range between the 25th and 75th quantiles; the line in the middle depicts the population median. Dashed vertical whiskers show the range. In all intervals except 4.5 cm, the f0 chamber mean is either the same or lower than the f1 mean. In the 4.5 cm interval, the f0 chamber mean is significantly higher than the f1 or f1 chamber mean. No significant differences were found between f1 and f2 chamber means.

We use LA-ICPMS to measure Mg/Ca on between 63 to 150 individual specimens of *T. sacculifer* from thirteen sediment intervals in two central tropical Pacific sediment cores (Fig. 1). We performed Mg/Ca analysis on the f0 and f1 chamber for all specimens, and on the f2 chamber from five sample intervals. Mg/Ca from each measured chamber are shown in Fig. 2.

We first assess differences in population-level metrics of the Mg/Ca values from all individuals for each chamber (summarized in Supplementary Table 1). Significant inter-chamber differences in mean Mg/Ca values are present. The f0 population mean Mg/Ca differs from f1 in 11 of 13 intervals (Fig. 2, Student’s t-test p < 0.05). In 10 of these 11 intervals, the f0 Mg/Ca mean was significantly lower than the f1 mean. In one sample (4.5 cm from 14MC1) this pattern is reversed, and the f0 mean is greater than the f1 mean (Student’s t-test, p < 0.05). Population mean Mg/Ca from the f0 chamber is lower than the mean of the f2 chamber measurements in three out of five intervals (Fig. 2). The 4.5 cm sample from 14MC1 again shows the opposite pattern, with the f0 mean higher than f2 (Student’s t-test, p < 0.05). We detect no difference in f1-f2 chamber Mg/Ca means in any of the five intervals with both f1 and f2 Mg/Ca results (Student’s t-test, p > 0.05).

**Figure 3:**
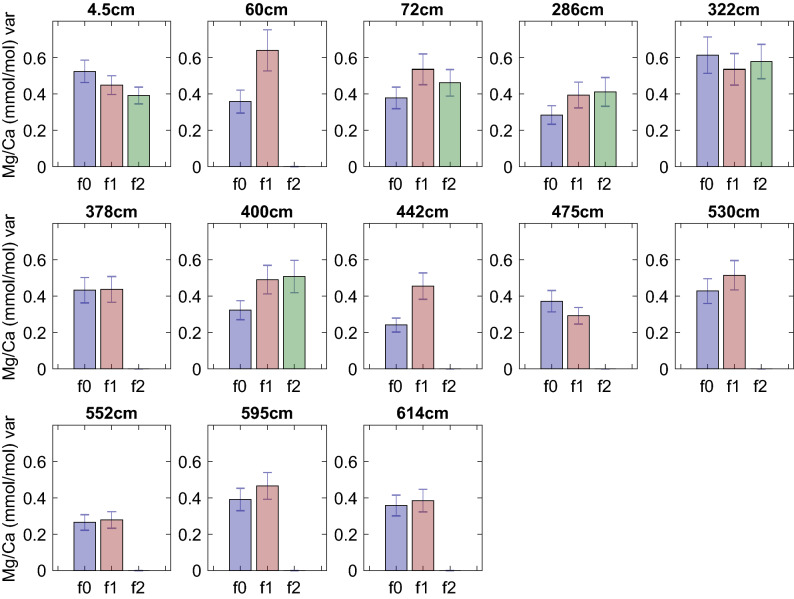
Variance of Mg/Ca values from each chamber at various sample depths. The error bars show the standard error of the variance. Significant differences in variability (one-sided f-test, p < 0.05) between f0 and f1 chambers are found at 60 cm, 400 cm and 442 cm, and between f0 and f2 at 400 cm. No intervals show significant differences between f1 and f2 chamber variability.

We assessed the differences in the dispersion of the Mg/Ca values by calculating the interquartile range, shown in Fig. 2, and the variance of all Mg/Ca values in each chamber, as shown in Fig. 3. We find significant differences in the variance of the f0 and f1 populations in three out of thirteen intervals (60 cm, 400 cm and 442 cm) (one-tailed f-test, p < 0.05). We detected a significant difference between f0 and f2 variability in the 400 cm interval (one-tailed f-test, p < 0.05), and no significant differences in variability were detected between the f1 and f2 chambers. However, as many of the population distributions are non-normal (based upon Anderson–Darling tests) we employ the Kruskall–Wallis test to determine if the individual-chamber distribution of Mg/Ca values in a sample are drawn from the same distribution. The null hypothesis is rejected at intervals 4.5 cm, 286 cm, 322 cm, 378 cm, 400 cm, 442 cm, 475 cm and 552c, 595 cm and 614 cm (p < 0.05), suggesting that the population distributions between chambers in these intervals differ. While these results may be sensitive to change in the median between chambers, they indicate that further investigation into the distribution of Mg/Ca values by chamber is warranted.

**Figure 4:**
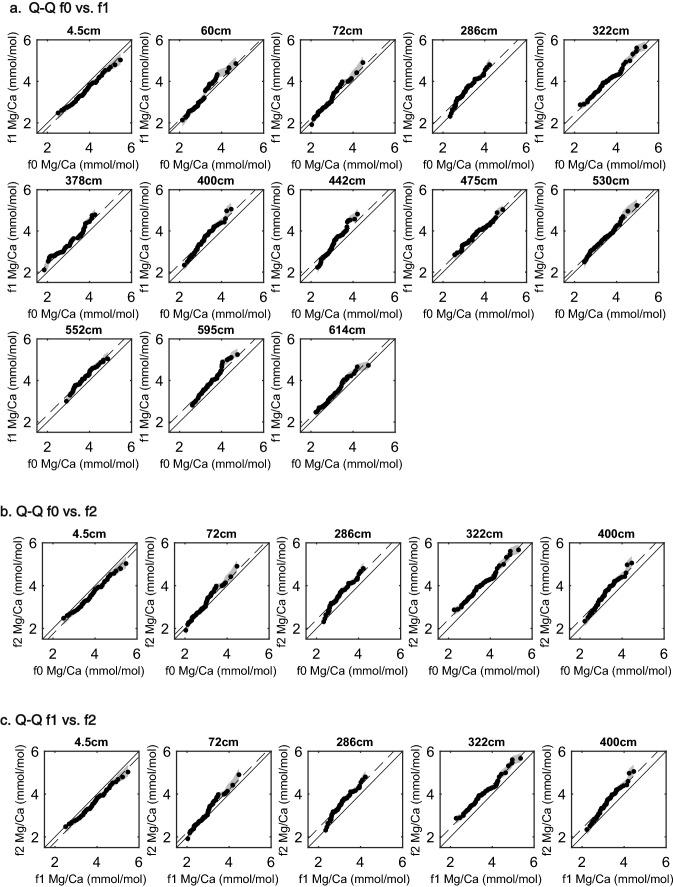
Quantile–quantile analysis of Mg/Ca values from different chambers. Identical distributions will fall along the 1:1 line (solid black diagonal), those that differ in mean will fall along the dotted line, which runs through the median of the y-axis population. Shaded regions depict the analytical uncertainty about the calculated quantiles. Population differences are largely observed at the low Mg/Ca ends of the populations.

Finally, we use quantile–quantile (Q–Q) analysis (see Methods) to assess how the distribution of Mg/Ca values differs within samples. In this analysis, identical populations will plot along a 1:1 line (solid diagonal), and populations that differ only in the mean will plot along a parallel line but offset (dashed line). We find that the populations of Mg/Ca values from each chamber are, in general, similar, aside from the previously described mean offsets (Fig. 4). Across all of the sample intervals, the distribution of Mg/Ca values in different chambers show little difference in the upper end of the Mg/Ca scale and fall on the 1:1 or offset 1:1 line within the LA-ICPMS analytical uncertainty (± 0.07 mmol/mol). Exceptions are found in the 442 cm and 595 cm intervals, where f1 high Mg/Ca quantiles are slightly elevated above the offset 1:1 line, and in the 400 cm interval, where the upper end of the f2 distribution is elevated above both the f0 and f1 chamber distributions. More substantive differences are observed at the lower end of the Mg/Ca scale. In the 60 cm, 72 cm, 286 cm, 400 cm, 442, 530 cm, and 595 cm interval, the low end of the f1 distribution is lower than the lower end of the f0 distribution. Lower f2 Mg/Ca, compared to both the f0 and f1 chambers, is observed in the 72 cm, 286 cm and 400 cm intervals. The overall differences are small (< ± 0.4 mmol/mol), but the impact may be magnified when converting these values to temperature.

### Analysis of between-chamber LA-ICPMS values

**Figure 5:**
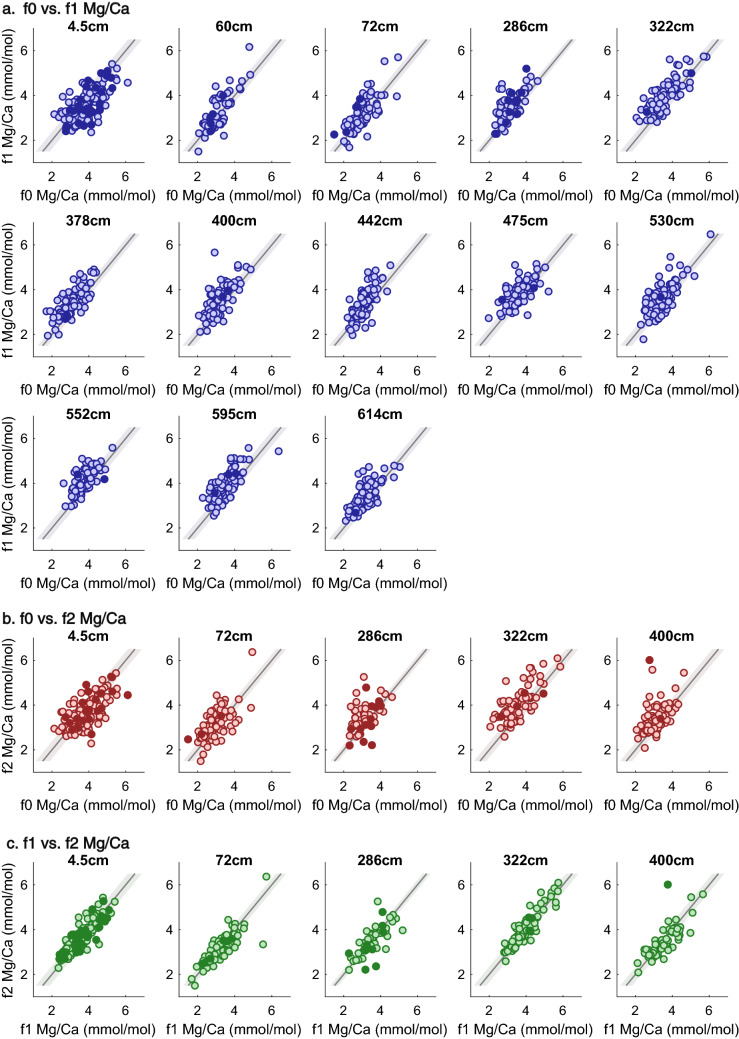
Chamber-to-chamber Mg/Ca differences in each individual foraminifer. Each point in the plots is one specimen, with the f0 (**a**, **b**) or f1 (**c**) chamber on the x-axis, and the f1 (**a**) or f2 (**b**, **c**) chamber on the y-axis. Individuals with the same mean Mg/Ca values in each chamber will fall along the 1:1 line (dashed black line). Points (individuals) that fall above the 1:1 line show higher Mg/Ca in the y-axis chamber, while points below the 1:1 line have higher Mg/Ca in the x-axis chamber. In a), the 4.5 cm shows more individuals below the 1:1 line, reflecting higher f0 vs f1 Mg/Ca values, compared to intervals with lower f0 vs. f1 Mg/Ca values. In (**c**), the correspondence between f1 and f2 Mg/Ca values is reflected in the close proximity of most individuals to the 1:1 line. The shaded region about the 1:1 line represents 2 × the average combined uncertainty for the chambers in the listed interval. Filled points show individual foraminifera with a single LA-ICPMS value for at least one of the chambers.

We now examine the differences in Mg/Ca values between chambers at the individual level by comparing different chambers on the same individual. In Fig. 5, each point represents an individual specimen and the Mg/Ca values from each measured chamber. Individuals closer to the 1:1 line have smaller chamber-to-chamber differences. In all intervals there are individuals with significant differences between the f0 and f1 chamber (i.e., unshared variance). In intervals with lower f1 mean Mg/Ca, more individuals show significantly reduced f1 Mg/Ca compared to f0 (combined analytical uncertainties are accounted for in quadrature). In these intervals 50% or more of individuals have f0 Mg/Ca values that are lower than their f1 or f2 Mg/Ca values, outside of the combined individual uncertainty. For the 14MC1 4.5 cm interval with higher mean f0 Mg/Ca compared to f1, 50% of individuals show increased Mg/Ca in their f0 chamber compared to their f1 chamber. Thus, the differences we observe in the mean chamber values at the population level are reflected at the individual level. However, considerable scatter is observed both within populations and among individuals. In all intervals, individuals with both higher and lower f0-to-f1 ratios occur, but the proportion of those individuals displaying such results varies, reflected in the population mean differences. In contrast, Mg/Ca in the f1 and f2 chambers (Fig. 5c) is more similar at the individual level. Compared to the f0-f1 individual differences, fewer individual f2-f1 values differ outside of the combined uncertainty (Supplementary Table 1).

### Comparison of LA-ICPMS and ICP-OES Mg/Ca data

**Figure 6:**
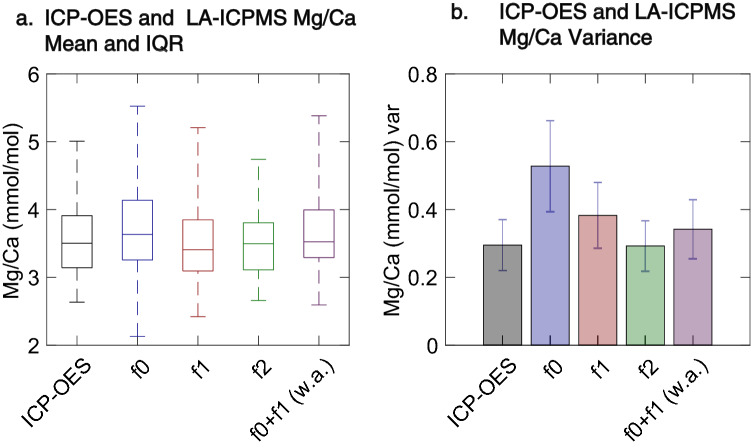
Comparison of same-individual Mg/Ca from ICP-OES and LA-ICPMS. (**a**) population mean, interquartile range, and range of Mg/Ca values from ICP-OES and LA-ICPMS analysis on the same individuals. LA-ICPMS results are shown separated by chamber (f0, f1, f2) and using the 55% f0 + 45% f1 weighted average (w.a.) that produces the maximal correlation. No statistical difference is observed in the population means (Student’s t-test, p > 0.05, N = 32). (**b**) population variance for ICP-OES and LA-ICPMS Mg/Ca, also separated by chamber and with the 55% f0 + 45% f1 weighted average. Error bars show the standard error of the variance. None of the variances differ significantly (one-sided f-test, p > 0.05).

**Figure 7:**
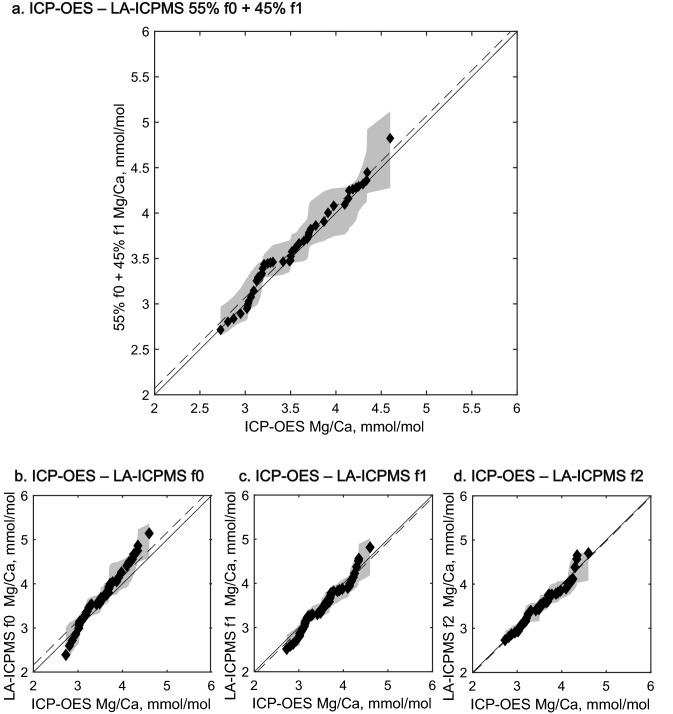
Quantile–quantile analysis of ICP-OES Mg/Ca and LA-ICPMS Mg/Ca. The population distribution of Mg/Ca values from ICP-OES is identical to the Mg/Ca values obtained using LA-ICPMS and the 55% f0 + 45% f1 weighted average maximal correlation, shown in (**a**). (**b**) The same analysis using LA-ICPMS Mg/Ca from the f0 chamber only. (**c**) The same analysis with only the f1 chamber, and (**d**) same analysis with only the f2 chamber. In all cases, the ICP-OES Mg/Ca and LA-ICPMS Mg/Ca distributions are highly congruent.

We have demonstrated that single specimens of *T. sacculifer* may exhibit significant inter-chamber variability. Such variations have the potential to alter measures of population distribution that are commonly used in single foraminifer palaeoceanographic reconstructions. To fully assess the impact of this intra-test heterogeneity at the individual level, we compared the LA-ICPMS data to Mg/Ca measured on the same shells after chemical cleaning and analysis via ICP-OES. We analyzed 32 individuals from the 14MC-1 4.5 cm sample which have data from the f0, f1 and f2 chambers interval using ICP-OES. Mg/Ca data from ICP-OES is a single, integrated value for the entire shell, excepting the ablated material and any material lost during cleaning, and thus our data enable us to test whether differences in chamber composition significantly impact population metrics and distributions. We find that Mg/Ca values measured on the same individual using LA-ICPMS and ICP-OES correspond at both the population and individual levels. The population mean of our ICP-OES Mg/Ca data (3.55 mmol/mol ± 0.10) does not differ significantly from the population mean of the LA-ICPMS data from the f0 (3.71 ± 0.13), f1 (3.49 ± 0.11) and f2 (3.53 ± 0.10) chambers (Student’s t-test, p < 0.05, n = 32) (Fig. 6a). We also find no difference in the variance of ICP-OES Mg/Ca data (Fig. 6b) compared to the f0, f1, and f2 chambers (one-sided f-test, p > 0.05), and nonparametric testing indicates the populations are likely drawn from the same distribution (Kruskall–Wallis test, p > 0.05). We next used Q-Q analysis to test the similarity of Mg/Ca distributions from ICP-OES and LA-ICPMS, as shown in Fig. 7. We find that the Mg/Ca distribution from ICP-OES is the same as that from LA-ICPMS of the f0, f1 and f2 chambers. At the population level, we conclude that the ICP-OES and LA-ICPMS Mg/Ca populations are statistically identical.

**Figure 8:**
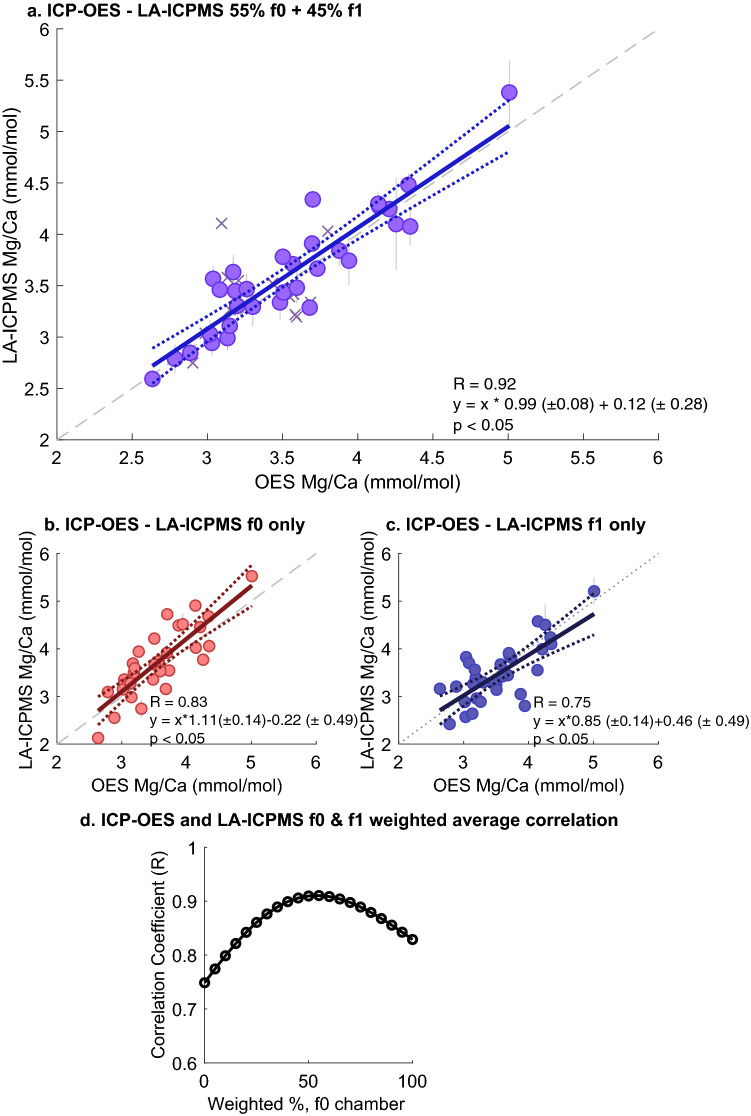
Correlation between ICP-OES and LA-ICPMS Mg/Ca values from paired analysis on the same individuals. Each point in **a-c** represents a single individual analyzed via both ICP-OES and LA-ICPMS. (**a**) The correlation between ICP-OES Mg/Ca values and the LA-ICPMS derived Mg/Ca using a weighted average of 55% f0 and 45% f1 chamber values (N = 32). (**b**, **c**) show the relationship between ICP-OES Mg/Ca and LA-ICPMS derived Mg/Ca using only the f0 or f1 chamber values. f2 chamber values (not shown) are similar to f0. (**d**) The correlation coefficient (R) obtained using different weightings of the f0 and f1 chamber. Maximal correlation (e.g., maximum R value, y-axis) occurs at 55% f0 (x-axis) and 45% f1 weighting.

We now investigate the relationship between paired ICP-OES and LA-ICPMS Mg/Ca at the individual level by directly comparing Mg/Ca values from each individual, as shown in Fig. 8. The correlation coefficients (R) for the individual chamber LA-ICPMS Mg/Ca values and ICP-OES Mg/Ca are 0.83 (f0), 0.75 (f1), and 0.76 (f2) (p < 0.05 for all relationships). Given the similarity of the population distributions, such correspondence is not surprising. However, in all cases the slope of the regression line differs from 1, and thus the relationship between the laser ablation and OES values is not 1:1, as expected if each method is measuring the same Mg/Ca value. Each individual chamber represents only a portion of the shell measured by ICP-OES. Therefore, we sought to calculate a weighted average of the individual chamber values that best reflects the whole-shell Mg/Ca value. We use only the f0 and f1 chamber, as the f1 and f2 chambers have been previously demonstrated to have similar Mg/Ca values. We find that the maximum R value of R = 0.92 is obtained when the f0 chamber is weighted at 55% and the f1 chamber at 45% (Fig. 8a). With this weighting, the slope of the line is 0.99 ± 0.08, providing a nearly 1:1 correspondence between ICP-OES and LA-ICPMS data. The improvement in the explained variance in the LA-ICPMS data is also substantial, increasing from 67% for the f1 chamber alone to 85% for the weighted average. Our assumption that the f2 chamber makes little difference is borne out in additional weighting tests, where no substantive improvement in this relationship is obtained with any f2 weighting. Similar results (e.g., a regression slope ~ 1 and a correlation coefficient above 0.91) are obtained when the relative weighting of the f2 chamber is low and the weighting of the f0 chamber is 50–55%. This finding demonstrates that LA-ICPMS data from the f0 and f1 chamber of *T. sacculifer* are sufficient to reproduce Mg/Ca data obtained from ICP-OES analysis of the entire specimen for this species. Q-Q analysis shows a close correspondence between ICP-OES and LA-ICPMS populations when this weighted averaging is applied (Fig. 7a), with no significant differences between quantiles in the populations and little offset in the population mean, indicating these populations are statistically identical.

## Discussion

### Inter-chamber differences

Our results present us with significant inter-chamber differences in the mean and variability of Mg/Ca, as well as differences in the distribution of those Mg/Ca values. This inter-chamber variability that we observe with LA-ICPMS may be due to factors such analytical uncertainty or differences in foraminifera life history. Microscopic variations in shell composition and analytical uncertainty associated with LA-ICPMS will result in some degree of within-chamber variability^10,11,29^ that translates to scatter in the relationships between chambers. These factors, while present, are likely not the primary cause of between chamber differences because we average several ablation sites from each chamber, reducing both the analytical uncertainty and the influence of microscopic variations in shell composition.

Migration of individual foraminifera in the water column before gametogenesis may explain the general decrease in Mg/Ca from the f2/f1 chambers to the final f0 chamber that we observe. Evidence of increasingly deeper habits through ontogeny has been inferred from detailed, chamber-by-chamber δ^18^O analysis^37^ and from previous analyses of foraminiferal growth patterns and habits^13,22^ for some species of foraminifera, including *T. sacculifer*. The standard models of chamber formation and growth postulate that calcite is added to the entirety of the test when new chambers are added, and this additional calcite represents a large proportion of the total calcite of the individual^33^. Thus, deeper and colder habitats may lead to the addition of lower Mg/Ca chambers, as well as lowering the mean Mg/Ca on existing chambers by its addition. In controlled laboratory conditions, ontogenetic effects have also been shown to reduce final chamber Mg/Ca compared to previous growth chambers^33^, which may also explain our general findings of lower f0 Mg/Ca. However, there is also evidence in foraminifera of diurnal banding of high and low Mg mediated by the activity of photosynthetic symbionts^13,31^. Thus, it is also possible that our observed inter-chamber variations in Mg/Ca reflect change in photosymbiont activity as a result changing light levels, possibly in response to water column migration.

It is likely that some combination of vertical migration (temperature and photosymbiont activity) and ontogenetic effects are responsible for the between chamber differences. In our sample intervals with lower f0 Mg/Ca, ontogenetic effects plus vertical migration can explain the chamber differences. In sample intervals that show no difference between f0 and f1 Mg/Ca, it is possible that changes in depth are insufficient to significantly alter temperature, possibly as a result of a deeper central equatorial Pacific mixed layer with a reduced thermal gradient. Conversely, it may indicate a stronger thermal gradient, narrower depth habitat, and/or reduced vertical migration. In the sample interval with higher Mg/Ca in the f0 chamber, it may be possible that light attenuation resulting from a deeper habitat resulted in higher-Mg calcite forming the bulk of the final f0 chamber, analogous to high-Mg banding observed in studies of diurnal Mg/Ca differences^13,31^. Further calibration studies investigating the effects of lower light levels on *T. sacculifer* would be needed to test this hypothesis. However, our results suggest that inter-chamber Mg/Ca differences may contain useful oceanographic and biological information.

### LA-ICPMS can provide whole shell Mg/Ca values

The strength of the correlation between ICP-OES solution chemistry whole-specimen Mg/Ca and LA-ICPMS Mg/Ca provides clues to the growth pattern of the foraminifer as well as guidance on the use of LA-ICPMS for palaeoceanographic reconstructions. The general assumption of individual foraminifera analysis is that the measured whole specimen Mg/Ca integrates the entire life history of the individual, providing 2–4 week ‘snapshots’ of ocean conditions. The correlation of such whole specimen Mg/Ca values from core tops with surface ocean temperature and variability suggests that this value is a representative proxy of past ocean temperatures^15,23^. Differences in population means, variances, and distributions between whole specimen Mg/Ca and individual-chamber (f0, f1, f2) Mg/Ca values demonstrate that individual-chambers do not fully capture the relevant whole specimen population statistics. Weighted averaging of the f0 and f1 chambers integrates more of the foraminifera’s life history and provides a better measure of the whole-specimen Mg/Ca value, regardless of whether the foraminiferal growth model assumes addition of calcite to existing layers or not. That the weighting between chambers is close to equal and does not need additional chambers (e.g., f2) suggests that the foraminiferal calcite added after chamber formation serves to reduce inter-chamber variability, such that the f1 and f2 chamber are statistically indistinguishable in most cases. Our results support foraminifera growth models that suggest that calcite is added to the entire specimen during the growth of the final chamber, and likely brings the Mg/Ca values of previous chambers closer to the value of the f0 chamber via its addition. Whether this happens on diurnal cycles or only during the growth of new chambers cannot be discerned by our data. However, it does re-enforce that both early and late ontogenetic calcite contributes to the whole-specimen Mg/Ca, and that such values should be treated as integrated signals over the lifetime of the individual.

The strong correlation that exists between individual paired ICP-OES and LA-ICPMS Mg/Ca values has implications for foraminiferal cleaning procedures for individual specimens. Chemical cleaning procedures, especially reductive and acid cleaning steps, can significantly alter Mg/Ca ratios obtained from either LA-ICPMS on individual specimens^38,39^ or from ICP-MS or ICP-OES analysis on aggregate samples^28,40,41^. The reductive cleaning and acid leaching steps are omitted in the single-specimen method of Rongstad et al.^15^, and cleaning is limited to water/methanol sonication and rinses, and heated oxidative cleaning with sonication. The close correspondence of our ICP-OES data with the same-specimen LA-ICPMS data suggest that the cleaning procedures employed here do not have significant impacts on the Mg/Ca values. It should be noted that the foraminifera from the central tropical Pacific analyzed in this study show little evidence of Mn contamination from LA-ICPMS data (see Methods). Al values, measured via OES, were below 4 ppm. These foraminifera also contained minimal residual sediment, and showed few visible signs of dissolution upon visual inspection. With these limitations and conditions in mind, the correspondence that we find between solution chemistry results and those from LA-ICPMS suggests that calibration equations generated from solution chemistry methods^4–6^ are applicable for use with LA-ICPMS data.

## Conclusion

The insight gained by trace element analysis by LA-ICPMS demonstrates that there are significant differences in the Mg/Ca values obtained from the three final growth chambers of *T. sacculifer* specimens from the central tropical Pacific Ocean. These differences may be attributable to differences in depth habitat and migration, ontogenetic factors, or photosynthetic symbiont activity, or most likely a combination of all three factors. Inter-chamber Mg/Ca differences may therefore contain useful oceanographic and biological information. The differences observed between chambers indicates that measurements of Mg/Ca from one chamber are not representative of whole-specimen Mg/Ca values, such as obtained from solution chemistry methods that involve dissolution of the entire specimen prior to analysis. Using paired ICP-OES and LA-ICPMS measurements on the same individual shells, we obtained a maximum correlation (R = 0.92) between Mg/Ca measured with each method using a weighted average of 55% f0 Mg/Ca and 45% f1 Mg/Ca. This robust correlation demonstrates that solution chemistry methods and LA-ICPMS methods are compatible, that cleaning procedures used on individuals are sufficient to obtain consistent results with other methods, and that calibration equations derived from solution chemistry-based experiments are applicable to LA-ICPMS data.

## Materials and methods

Piston core ML1208-17PC (hereafter 17PC) was recovered at 0.48°N, 156.45°W, at a depth of 2926 m, and multicore ML1208-14MC1 (hereafter 14MC) at 0.22°S, 155.96°W, from 3049 m water depth (for location map, see Supplementary Fig. S1). Specimens of the planktic foraminifera *T. sacculifer* without the final sac were picked from 1-cm intervals beginning at 4.5 cm of 14MC and the 60 cm, 72 cm, 286 cm, 322 cm, 378 cm, 400 cm, 442 cm, 475 cm, 530 cm, 552 cm, 595 cm, and 614 cm intervals of 17PC. Sediments in the 4.5 cm interval have been radiocarbon dated to 3.4 ky^8^, and 17PC sediments are from 24 to 282 ky^9^. Individuals were selected from the 355–425 μm size fraction to reduce ontogenetic effects^42^. Specimens were sonicated in deionized water to remove loose surface materials and dried in a 55 °C oven, then washed in ethanol and/or methanol.

Individual specimens of *T. sacculifer* were first analyzed for trace metals via laser ablation inductively coupled mass spectrometry (LA-ICPMS). LA-ICPMS trace metal analysis was performed on a Photon Machines Analyte.193 with HelEx sample cell with a Thermo ElementXS inductively coupled plasma mass spectrometer. Individuals were affixed to scanning electron microscope tape with the main aperture of the final chamber (f0) facing upwards, exposing the outer surface of the three final chambers (f0, f1, and f2). Three to five 50 μm laser ablation locations were targeted on the f0 chamber, and two to three 50um targets were selected on both the f1 and f2 chambers (Fig. 1). Laser ablation power of 1.2 J/m^2^ was used to obtain 1–4 min of ablation time at 4 Hz from the outside of each chamber until breaking through the inside chamber wall. Mg/Ca ratios were calculated using automated functions from raw output of elemental counts/s calibrated to NIST 610. Ablation profiles are truncated at both the beginning and end of ablation to remove the influence of surface coatings, and a buffer was applied both ends of the data acquisition to remove residual data. The uncertainty of the mean for each chamber calculation is the standard error of the mean (σ/√n)^43^. Overall standard error of Mg/Ca measurements on the f0 chamber is ± 0.065 mmol/mol, and on the f1 and f2 chambers is ± 0.074 mmol/mol. The concentration of Mn was monitored to detect Mn-coatings or nodules and samples were removed if Mn exceeded 1200 counts/s, as such contamination can bias measured Mg^26^. Specimens for same-shell LA-ICPMS/ICP-OES intercomparison were carefully removed from the SEM tape using methanol and ethanol. Tape residue was removed by repeated brushing with methanol until tape residue was no longer visible. Individuals were then gently cracked to open the chambers and subject to chemical cleaning procedures following those outlined in Rongstad et al., including clay removal by ultrasonication in ultra-pure water, methanol rinses, and heated oxidative cleaning using H_2_0_2_ in an NaOH solution. Trace element-to-Ca ratios (including Mg/Ca) were measured for each specimen via inductively-coupled plasma optical emission spectrometry (ICP-OES) using a Thermo Fisher Scientific iCAP 6500 duo with a Cetac 520 autosampler at the Lamont-Doherty Earth Observatory. Concentrations of Al were monitored to indicate the presence of remnant clay minerals and as an indicator for the presence of residual SEM tape, which was found to be rich in Al during laser ablation tests. ICP-OES calibration was performed using a seven-point linear dilution including a laboratory blank and standards ranging from 1.5 to 15.1 ppm Ca and 4.0 to 38.8 ppb Mg. Dilutions of the calibration standard were run at 16, 10, 5, 2.5, 1.25 and 0.625 ppb Mg to determine the concentration below which reliable Mg results no longer occur. Repeated testing of low-concentration standard solutions showed an increase in RSD and variability below 5 ppb Mg, and we used this as the lower limit for further analysis. Mg concentrations above 5 ppb have standard deviations below ± 0.1 ppb, with resulting Mg/Ca ratios within 0.1 mmol/mol of the standard.

Consistency standards with 3.1 ppm Ca and 9.1 ppb Mg were run every other sample and Mg/Ca values were drift corrected to these standards. Repeatability of the consistency standard (Mg/Ca) is 4.86 ± 0.037 mmol/mol (0.75% RSD). Check standards were run at ~ 9.5 ppb Mg and a Mg/Ca ratio of 2.67 mmol/mol with an overall standard deviation of ± 0.046 mmol/mol.

### Individual and population-level Mg/Ca values

Mg/Ca ratios for each chamber are reported as the mean of all targeted profiles on that chamber. Uncertainty for each chamber is the standard error of the mean (SEM). Population mean refers to the mean value of all of the calculated individual chamber Mg/Ca values for a given chamber in each sample interval. Population variance refers to the variance of all of the individual Mg/Ca values obtained from a given chamber. Individual-level chamber differences refers to the difference between Mg/Ca value from one chamber (as calculated above) and another chamber from a single individual. Values that are considered statistically different are outside of the uncertainty (SEM) for both chambers. For chambers with a single measurement, the average uncertainty for that chamber and that interval is used in determining statistical difference.

### Quantile–quantile analysis

We perform quantile–quantile (Q–Q) analysis to detect differences in the population distributions of Mg/Ca values obtained from individual chamber values using methods adapted from those previously described and used for assessing population differences in individual foraminifera populations^7–9^. In our analyses, we calculate an empirical cumulative distribution function (ECDF) for both the reference (X-axis) and test (Y-axis) populations. We then calculate the quantiles for the populations from these ECDFs. Uncertainty about the quantiles for the test population are calculated using the randomized analytical uncertainty for that test population. Results are displayed plotting the relationship of the reference population (X-axis) versus the sample population and confidence intervals (Y-axis). Populations which do not statistically differ fall along a diagonal 1:1 line (e.g., x = y) within the confidence interval bounds. Populations that differ only in mean will be offset from this 1:1 line on a line parallel to it with a slope of 1 (shown as a dotted line in our plots). Relative distribution differences are determined by correspondence with this offset line. Differences in population distributions are considered significant when the confidence interval about a quantile does not include the offset 1:1 line.

### Statistical analysis

All test statistics were calculated using MATLAB 2019a. Correlation coefficients (R and R^2^), slopes, intercepts and p-values for statistical significance were determined using MATLAB 2019a’s ‘fitlm’ linear modeling function.

## Supplementary Information

Supplementary Dataset.

Supplementary Information 1.

## Data Availability

Data for this study is available in the supplemental data file Supplementary_Dataset1.xlsx.
