# Supplementary material for: Relationship between individual chamber and whole shell Mg/Ca ratios in Trilobatus sacculifer and implications for individual foraminifera palaeoceanographic reconstructions
Source: Sci Rep. 2021 Jan 11;11:463. doi: 10.1038/s41598-020-80673-8 (PMC7801486; doi:10.1038/s41598-020-80673-8)
Supplement: Supplementary file 2 — Supplementary Information 1. [file 41598_2020_80673_MOESM2_ESM.pdf]

Supplementary Information for Relationship between individual chamber and whole shell Mg/Ca ratios in *Trilobatus sacculifer* and implications for individual foraminifera palaeoceanographic reconstructions

Rustic, Gerald T. * [1,2] , Polissar, Pratigya J. [3], Ravelo, Ana Christina [3], DeMenocal, Peter [4,^2]

[1] Department of Geology, School of Earth and Environment, Rowan University, Glassboro, NJ 08028

[2] Lamont-Doherty Earth Observatory, Columbia University, Palisades, NY 19604

[3] Ocean Sciences Department, University of California at Santa Cruz, Santa Cruz, CA 95064

[4] Woods Hole Oceanographic Institution, Woods Hole, MA, 02543

[^] formerly at [2]

* corresponding author, rustic@rowan.edu

Table of Contents

Supplementary Figures 2

Supplementary Tables 3

Supplementary References 4

Supplementary Figures

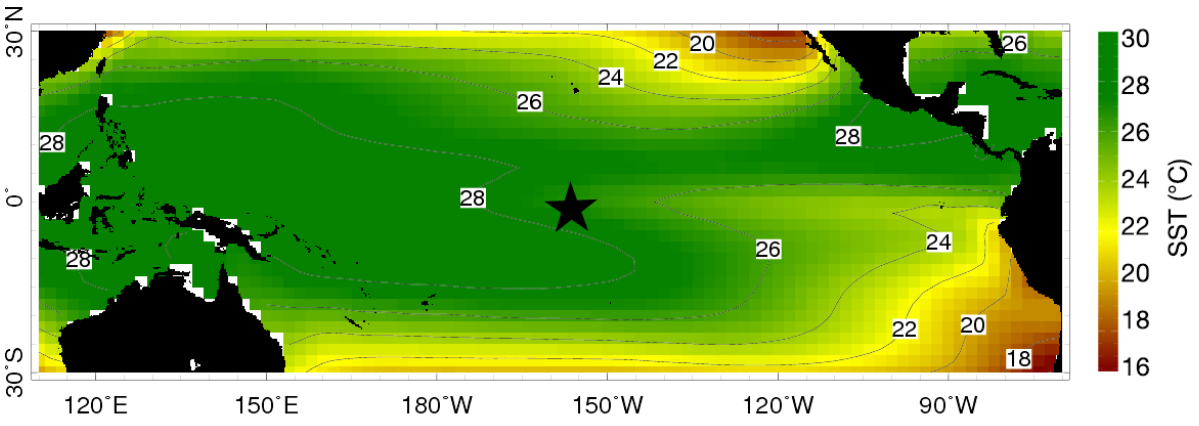

Supplementary Figure 1. Map of the tropical Pacific Ocean showing study location and average sea surface temperature. Star shows the location of ML1208 17PC (0.48°N, 156.45°W, 2926m depth) and ML1208 14MC1 (0.22°S, 155.96°W, 3049m depth) used in this study. Average sea surface temperature from the ERSSTv5 1854-2020 ([1]).

Supplementary Tables

Core / Depth	Chamber	N	Norm	Mg/Ca Mean (mmol/mol)	Mg/Ca Variance (mmol/mol)	Chambers	Chamber-to-Chamber diff.
14MC1 4.5cm	f0	150	0	3.81 ± 0.06	0.52 ± 0.06	f0-f1	56.67% (24.00%)
	f1	150	1	3.56 ± 0.05 *	0.45 ± 0.05	f0-f2	52.78% (28.47%)
	f2	144	1	3.64 ± 0.05 *	0.39 ± 0.05	f1-f2	26.39% (50.69%)
17PC 60cm	f0	65	1	3.06 ± 0.07	0.36 ± 0.06	f0-f1	33.85% (43.08%)
	f1	65	1	3.20 ± 0.10	0.64 ± 0.11 *	f0-f2	-
17PC 72cm	f0	82	1	2.98 ± 0.07	0.38 ± 0.06	f0-f1	28.05% (58.54%)
	f1	82	0	3.20 ± 0.08 *	0.53 ± 0.08	f0-f2	27.16% (53.09%)
	f2	81	1	3.14 ± 0.08	0.46 ± 0.07	f1-f2	43.21% (40.74%)
17PC 286cm	f0	63	1	3.15 ± 0.07	0.28 ± 0.05	f0-f1	17.46% (77.78%)
	f1	63	0	3.56 ± 0.08 *	0.39 ± 0.07	f0-f2	23.64% (61.82%)
	f2	55	0	3.51 ± 0.09 *	0.41 ± 0.08	f1-f2	38.18% (40.00%)
17PC 322cm	f0	77	0	3.59 ± 0.09	0.61 ± 0.10	f0-f1	16.88% (74.03%)
	f1	77	1	4.01 ± 0.08 *	0.53 ± 0.09	f0-f2	15.79% (69.74%)
	f2	76	1	4.06 ± 0.09 *	0.58 ± 0.09	f1-f2	26.32% (39.47%)
17PC 378cm	f0	79	0	3.03 ± 0.07	0.43 ± 0.07	f0-f1	11.39% (68.35%)
	f1	79	1	3.41 ± 0.07 *	0.44 ± 0.07	f0-f2	-
17PC 400cm	f0	79	0	3.20 ± 0.06	0.32 ± 0.05	f0-f1	12.66% (73.42%)
	f1	79	0	3.59 ± 0.08 *	0.49 ± 0.08	f0-f2	25.76% (60.61%)
	f2	66	1	3.49 ± 0.09 *	0.51 ± 0.09	f1-f2	48.48% (27.27%)
17PC 442cm	f0	80	0	3.19 ± 0.06	0.24 ± 0.04	f0-f1	17.50% (68.75%)
	f1	80	0	3.48 ± 0.08 *	0.46 ± 0.07 *	f0-f2	-
17PC 475cm	f0	82	0	3.62 ± 0.07	0.37 ± 0.06	f0-f1	19.51% (59.76%)
	f1	82	0	3.85 ± 0.06 *	0.29 ± 0.05	f0-f2	-
17PC 530cm	f0	82	0	3.47 ± 0.07	0.43 ± 0.07	f0-f1	28.05% (51.22%)
	f1	82	0	3.66 ± 0.08	0.51 ± 0.08	f0-f2	-
17PC 552cm	f0	78	0	3.76 ± 0.06	0.26 ± 0.04	f0-f1	10.26% (70.51%)
	f1	78	0	4.10 ± 0.06 *	0.28 ± 0.04	f0-f2	-
17PC 595cm	f0	81	0	3.52 ± 0.07	0.39 ± 0.06	f0-f1	16.05% (74.07%)
	f1	81	0	3.92 ± 0.08 *	0.47 ± 0.07	f0-f2	-
17PC 614cm	f0	78	0	3.15 ± 0.07	0.36 ± 0.06	f0-f1	21.79% (58.97%)
	f1	78	1	3.39 ± 0.07 *	0.38 ± 0.06	f0-f2	-

Supplementary Table 1. Population Mg/Ca data from each measured chamber of *G. sacculifer* from cores 14MC1 and 17PC. N is the number of individuals with measurements

from a given chamber. AD Norm is the results of the Anderson-Darling test for normality for the population for each chamber (1 indicates the population is normally distributed). Population mean Mg/Ca (in mmol/mol) is shown with the standard error. * denotes values that statistically differ from the f0 chamber mean in that interval (Student's t-test, $p<0.05$). Population variance is shown with the standard error of the variance. ^ denotes values that are significantly different than the f0 population variance for that interval (one-sided f-test, $p<0.05$). Chamber-to-chamber differences shows the percentage of individuals that have higher (lower) Mg/Ca values in the f0 chamber compared to f1, f0 compared to f2, and f1 compared to f2, outside of the combined uncertainty for each chamber.

Supplementary References

1. Huang, B., Thorne, P.W., Banzon, V.F., Boyer, T., Chepurin, G., *et al.* NOAA Extended Reconstructed Sea Surface Temperature (ERSST), Version 5. (2017) doi:10.7289/V5T72FNM.
